# Optogenetic Measurement of Presynaptic Calcium Transients Using Conditional Genetically Encoded Calcium Indicator Expression in Dopaminergic Neurons

**DOI:** 10.1371/journal.pone.0111749

**Published:** 2014-10-31

**Authors:** Carmelo Sgobio, David A. Kupferschmidt, Guohong Cui, Lixin Sun, Zheng Li, Huaibin Cai, David M. Lovinger

**Affiliations:** 1 Transgenics Section, Laboratory of Neurogenetics, National Institute on Aging, National Institutes of Health, Bethesda, Maryland, United States of America; 2 Laboratory of Integrative Neuroscience, National Institute on Alcohol Abuse and Alcoholism, National Institutes of Health, Rockville, Maryland, United States of America; 3 Unit on Synapse Development and Plasticity, National Institute of Mental Health, National Institutes of Health, Bethesda, Maryland, United States of America; University of Wurzburg, Germany

## Abstract

Calcium triggers dopamine release from presynaptic terminals of midbrain dopaminergic (mDA) neurons in the striatum. However, calcium transients within mDA axons and axon terminals are difficult to study and little is known about how they are regulated. Here we use a newly-developed method to measure presynaptic calcium transients (PreCaTs) in axons and terminals of mDA neurons with a genetically encoded calcium indicator (GECI) GCaMP3 expressed in transgenic mice. Using a photomultiplier tube-based system, we measured electrical stimulation-induced PreCaTs of mDA neurons in dorsolateral striatum slices from these mice. Single-pulse stimulation produced a transient increase in fluorescence that was completely blocked by a combination of N- and P/Q-type calcium channel blockers. DA and cholinergic, but not serotoninergic, signaling pathways modulated the PreCaTs in mDA fibers. These findings reveal heretofore unexplored dynamic modulation of presynaptic calcium in nigrostriatal terminals.

## Introduction

The striatum has the richest DA innervation in the CNS, provided by the widely arborized axons of mDA neurons [1]. Dopamine regulates neuronal function at both pre- and postsynaptic loci [2], and disruption of nigrostriatal DA signaling contributes to basal ganglia circuit dysfunction and psychomotor disorders, including Parkinson’s disease [3], [4]. Although calcium (Ca^2+^) is required for dopamine release [5], little is known about mechanisms and modulation of presynaptic Ca^2+^ dynamics in striatal DA terminals.

The ability to measure Ca^2+^ in DA terminals has been hampered by the small dimensions of DA axons and terminals, the massive distribution of DA axonal ramifications in the striatum [1], and difficulties associated with loading Ca^2+^ indicators selectively into DA axons/terminals while avoiding nearby cellular elements. Genetically Encoded Calcium Indicator (GECIs) based on chimeric fluorescent proteins allow investigators to avoid many limitations of conventional small-molecule Ca^2+^ dyes [6]. They can easily be targeted for expression in specific cell types, avoiding indiscriminate loading. Genetic expression also avoids dye injection that can damage tissues [6]. In particular, GCaMP3 has been used to examine neuronal Ca^2+^ transients in brain slices and *in vivo*, following infection with adeno-associated virus or transgenic expression [7]. However, the utility of GCaMPs for measuring presynaptic Ca^2+^ has not been widely assessed [8], [9].

Recently, expression of transgenic GCaMP3 and Ca^2+^ signaling in mice was characterized in various neuronal subpopulations using expression under the control of the Thy1 promoter, with a focus on postsynaptic calcium transients [10]. The sensor detected action potential bursts with good response linearity and photostability. Owing to these features, we selected GCaMP3 for the generation of a new line of transgenic mice, which express GCaMP3 in mDA neurons using a binary tetracycline-dependent inducible gene expression system [11], [12]. The expression of GCaMP3 selectively in the axons and axon terminals of mDA neurons in the striatum allowed us to systematically examine the dynamics of presynaptic Ca^2+^ transients in mDA neurons under various conditions. Our findings reveal the role of specific voltage-gated Ca^2+^ channels in Ca^2+^ entry into presynaptic elements of these neurons, and potent and selective modulation of presynaptic Ca^2+^ by dopaminergic and cholinergic receptor signaling.

## Materials and Methods

### Ethics Statement

All mouse work follows the guidelines approved by the Institutional Animal Care and Use Committees of the National Institute of Child Health and Human Development, US National Institutes of Health. The research was approved by the Animal Care and Use Committee of the Division of Intramural Clinical and Biological Research, National Institute on Alcohol Abuse and Alcoholism, NIH.

### Generation of Transgenic Mice

To develop a conditional GCaMP3 transgenic mouse model, the cDNA fragment encoding GCaMP3 (Addgene Plasmid 26974) [13] was inserted into the mouse prion protein (pPrP)–tetO gene expression vector (a gift from Dr. David Borchelt, University of Florida, Gainesville, FL), which is controlled by the tetracycline-responsive promoter (tetP) [14]. The tetO-GCaMP3 expression construct was then purified and microinjected into fertilized oocytes derived from C57BL/6J mice. The founder mice were crossed with wild-type C57BL/6J mice to produce the F1 generation.

PITX3/IRES2-tTA mice were obtained as previously reported [12]. PITX3 is predominantly expressed in mDA neurons, where it is involved in developmental processes, cell-specific gene expression and regulation of dopaminergic neurons [15]. PITX3/IRES2-tTA mice with 95% C57BL/6J strain background were crossbred with tetO-GCaMP3 transgenic mice in C57BL/6J strain background. Mice were housed in a 12-h light/dark cycle and fed regular diet ad libitum. Generation of D1-CRE/GFP mice was previously described [16].

### Genotyping

Genomic DNA was prepared from tail biopsy using DirectPCR Lysis Reagent (Viagen Biotech) and subjected to PCR amplification using specific sets of PCR primers for each genotype, including PITX3/− transgenic mice (PITX3-F, GACTGGCTTGCCCTCGTCCCA; PITX3-R, GTGC ACCGAGGCCCCAGATCA) and −/GC mice (GCaMP-F, TACTGCTCCATTTTGCGTGA; GCaMP-R, TTGCTGTCCACCAGTCATGC).

### Behavior Tests

Accelerating rotarod test. As described previously [12], [17], mice were placed onto a rotating rod with auto-acceleration from 0 to 40 rpm for 1 min (San Diego Instruments, San Diego, CA). The length of time the mouse stayed on the rotating rod was recorded, across 10 trials.

Open-field test. As described previously [17], the ambulatory and rearing activities of mice were measured by the Flex-Field activity system (San Diego Instruments, San Diego, CA). Flex-Field software was used to trace and quantify mouse movement in the unit as the number of beam breaks per 25 min. All behavioral results were compared using two-way ANOVA.

### Slice Preparation and Recordings

Following isoflurane anesthesia, brains from 10–14 weeks mice were removed and 250-µm-thick coronal sections through the striatum were prepared in carbogen-bubbled, cold high-sucrose solution (in mM: Sucrose 194, NaCl 30, KCl 4.5, MgCl_2_ 1, NaHCO_3_ 26, NaH_2_PO_4_ 1.2, Glucose 10) [18]. Slices were then transferred to a chamber filled with oxygenated artificial cerebrospinal fluid (aCSF; in mM: 124 NaCl, 4.5 KCl, 1.2 NaH_2_PO_4_, 2 CaCl_2_, 1 MgCl_2_, 26 NaHCO_3_, 10 glucose; in Ca^2+^-free aCSF, MgCl_2_ was increased to 3 mM) at 32°C and allowed to recover for 1 h. Slices were then pre-incubated at room temperature until used for recording.

### Photometric Recording

Hemisections were transferred to a recording chamber and constantly superfused with aCSF at 29–31°C at a rate of 1.5 ml/min using a peristaltic pump. A bipolar concentric electrode was positioned in the corpus callosum. Calcium transients were recorded in the dorsolateral stratum using a 40x/0.8 N.A. water-immersion objective on a Zeiss microscope (Carl Zeiss Microscopy GmbH, Jena, Germany). Fluorescence in regions of interest (ROI: 180 µm ×180 µm in a 250-µm thick slice) was excited using light emitted by a mercury burner (Zeiss FluoArc Variable Intensity Lamp Control for HBO 100, Carl Zeiss Microscopy GmbH, Jena, Germany) and attenuated to 35% brightness. A shutter (model V25; Uniblitz, Vincent Associates, Rochester, NY), with exposure controlled by a driver under TTL control (model D122, Uniblitz), was used to reduce exposure time and photobleaching. Ca^2+^ transients were evoked from populations of axon fibers within the ROIs considered, by rectangular, electrical pulse stimulation (120 µA, 10 ms, monophasic, unless otherwise noted) using an isolated constant current stimulator (DS3/GG2A system Digitimer Ltd, Hertfordshire, UK). Excitation exposures of 5-sec duration were recorded every 30 sec. Slices were allowed to sit in the recording chamber with afferent stimulation for 20 min before recording session began. The light emitted from the ROI was filtered at 535 nm and sent to a photomultiplier tube (PMT, model C6271; Hamamatsu Photonic Systems, Bridgewater, NJ). The PMT voltage output (time constant: 5 ms; gain: 400 × 10-1 µA/V) was fed into a computer interface (Digidata 1322A, Axon Instruments, Molecular Devices LLC, Sunnyvale, CA). Data were sampled at 100 Hz, and stored on a PC hard drive using PCLAMP 9.2 software (Axon Instruments, Molecular Devices LLC, Sunnyvale, CA). Analysis of transients was performed offline using cursor-based measurements in Clampex. Five sweeps evoked over each 5-min period were averaged, and corrected for gradual reductions in background fluorescence (most likely due to photobleaching and/or loss of signal from a small subpopulation of axons), using linear regression calculated from fluorescence measures with the stimulus-induced transient period removed (see Figure S1 in File S1). A 2-sec period was selected from the original averaged sweep and the peak of the time period containing the stimulus-induced transient was deleted and linear regression was performed on the remaining data points. A linear function was calculated and values at each point on this line were estimated. Each point in the original raw exported data was divided by the fit-generated estimated value at the same time point to obtain the normalized baseline value across the 2-sec period originally exported. Transients were measured as the ratio of the peak amplitude of the transient (ΔF) to the averaged baseline value (F) measured before the stimulus.

For pharmacological experiments, fluorescent transients were expressed as a percentage of the average of the first 10 min pre-drug control baseline, and compared to non-treated conditions, to account for the slight time-dependent decrease in the amplitude of the transient in the absence of treatment. Two-way ANOVA and MANOVA were used for statistical comparisons between different conditions. For better representation of the data, Bonferroni’s Post Hoc tests showed in Figures were applied to data collected during drug treatments, with the only exception being experiments with limited-duration exposure to Ca^2+^-free aCSF and experiments involving scopolamine treatment. Pearson r was used to calculate correlations between time constants and amplitude of the fluorescent transients.

### Fast-Scan Cyclic Voltammetry

Cylindrical carbon-fiber microelectrodes (50–100 µm of exposed fiber) were prepared with T650 fibers (6 µm diameter; Goodfellow, Coraopolis, PA) and inserted into a glass pipette (Cahill et al., 1996). The carbon-fiber electrode was held at −0.4 V, and the potential was increased to 1.2 V and back at 400 V/s every 100 ms using a triangle waveform. Dopamine release was evoked by rectangular, electrical pulse stimulation (120 µA, 10 ms, monophasic, unless otherwise noted) applied every 3 min. Data collection and analysis were performed using the Demon Voltammetry and Analysis software suite [19]. Ten cyclic voltammograms of charging currents were recorded as background before stimulation, and the average of these responses was subtracted from data collected during and after stimulation. The maximum amplitudes of extracellular DA transients were obtained from input/output (I/O) curves. I/O curves were constructed by plotting stimulus current versus concentration of DA response amplitude over a range of stimulus intensities. Two-way ANOVA were used for statistical comparisons between groups.

### Extracellular Field Potential Recording

Extracellular field recordings were acquired from 250 µm-thick coronal brain slices using glass recording electrodes filled with 0.9% NaCl solution (wt/vol). Population spikes ranging in amplitude from 0.6 to 1.4 mV were elicited at 0.033 Hz in the DLS by electrical stimulation (0.8–1.0 mA, 40 µs) from a concentric bipolar electrode placed just ventral to the overlaying white matter of the external capsule. Recordings were filtered at 1 kHz and digitized at 6.67 kHz using Clampex 9.2.

### Simultaneous Photometric and Voltammetric or Field Potential Recordings

For FSCV/photometry and extracellular field potential/photometry simultaneous recordings, two systems were synchronized to the same electrically evoked event for recordings. In the FSCV/photometry double recording, the intensity of the light was lower than the standard protocol (from 35% to 20%), to minimize the interference described in Figure S1 in File S1. To synchronize the two independent systems, Demon software was used to start the cyclic scanning together with a TTL signal for triggering the Clampex recording protocol. Subsequently, Clampex controlled both shutter status and electrical stimulation delivered through the isolated stimulator unit. For extracellular field potential/photometry simultaneous recordings, note that the frequencies of acquisition rate was increased from 100 Hz to 6.67 kHz, to be able to record the faster field potential events.

### Drugs

Cadmium chloride, ω-conotoxin GVIA (CTX), ω-agatoxin IVA (ATX), tetrodotoxin (TTX), Bay K 8644, nifedipine, quinpirole, sulpiride, CP-93,129, (2R)-amino-5-phosphonovaleric acid (APV), 2,3-dihydroxy-6-nitro-7-sulfamoyl-benzo[f]quinoxaline-2,3-dione (NBXQ), picrotoxin and citalopram were obtained from Sigma-Aldrich. Oxotremorine-m (OXO-M), dihydro-β-erythroidine (DHβE), and scopolamine were obtained from Tocris Cookson. Drugs were dissolved as stock solutions in water or DMSO and aliquoted and frozen at −20°C before use. Each of the drugs was diluted in aCSF immediately before each experiment. When used, the final concentration of DMSO external solution was always <0.05%.

### Immunohistochemistry

For basal localization of tyrosine hydroxylase (TH) and GFP expression, mice were perfusion fixed with 4% formaldehyde (PFA) and 40-µm coronal and sagittal sections were prepared as described previously [20]. Slices were fixed overnight with 4% formaldehyde in PBS, beginning at room temperature for at least 30 min and then sections were transferred to 4-°C. After washing overnight in PBS plus 0.2% triton X-100 (PBST), slices were rinsed in deionized water (dH_2_O), then incubated twice for 10 min in freshly prepared sodium borohydride (5 mg/m: in dH_2_O). After rinsing again with PBST, the slices were incubated for at least 4 h in 5% BSA in PBST. Antibodies specific to green fluorescent protein (GFP; 1∶2000; Sigma-Aldrich) and, Tyrosine Hydroxylase (TH; 1∶2000, Dynal Biotech) were used for incubation for 48 h at 4°C. After three rinses in PBS, tissue was incubated with 488- or 568-conjugated secondary antibodies (1∶500; Invitrogen) for 24 h. Fluorescence images were captured using a stereoscope (SteREO discovery, Zeiss, Thornwood, NJ) and a laser scanning confocal microscope (LSM 510; Zeiss, Thornwood, NJ). The stereoscopic images were collected using light emitted by a mercury burner filtered at 535 nm set at 12x magnification, using the same gain, focus from the surface and offset settings for each slice using AxioVision software (Rel. 4.8., Zeiss, Thornwood, NJ). The confocal images were collected using LSM imager or Leica LCS software, as single optic layer with a 100x/1.4 NA oil immersion objective. Excitation filter of 480/35 and emission filter of 535/30 were used for green fluorescence, while excitation filter of 540/25 and emission filter of 605/55 were used for red fluorescence. Postcollection processing was applied uniformly to all paired images using ImageJ (http://imagej.nih.gov).

## Results

### Generation and Characterization of GCaMP3 Inducible Transgenic Mice

**Figure 1:**
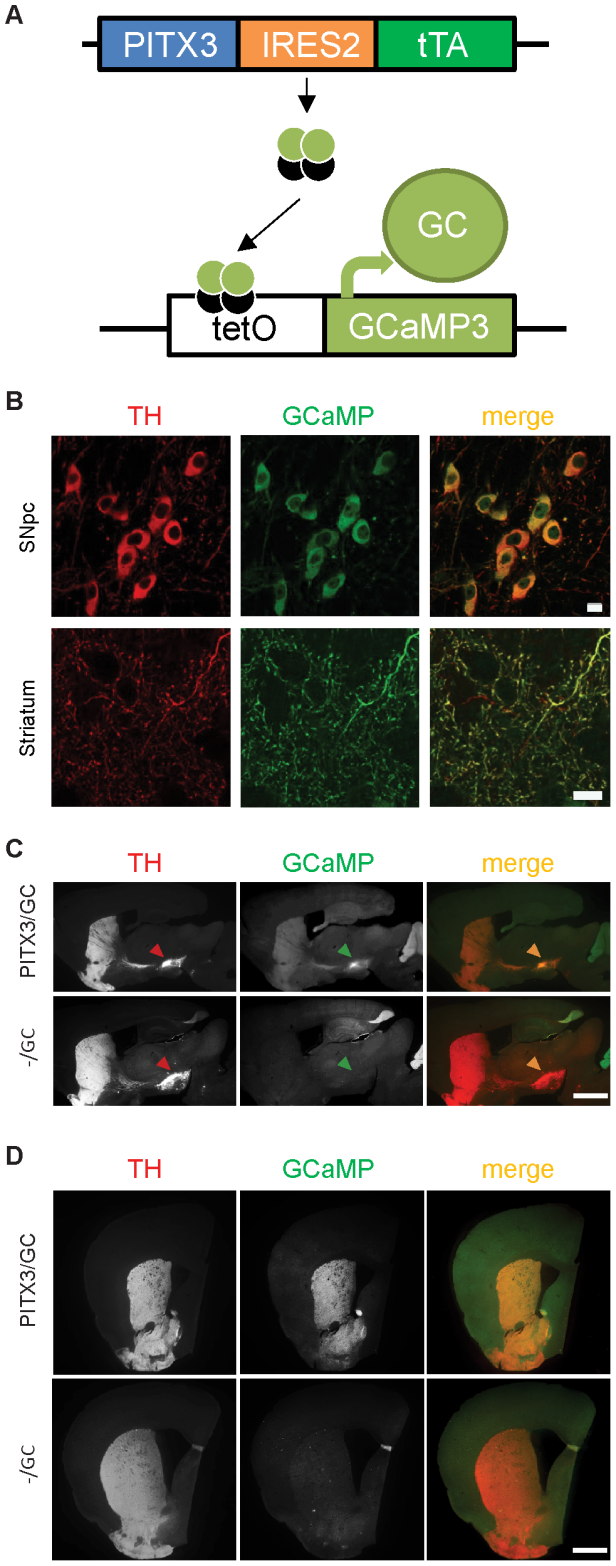
Generation of GCaMP3 conditional transgenic mice. (A) Schematic depicts the generation of PITX3-IRES2-tTA/tetO-GCaMP3 (PITX3/GC) double-transgenic mice. (B) Sample images of immunohistochemistry for tyrosine hydroxylase (TH) and the GFP moiety of GCaMP show GCaMP3 distribution in the dorsolateral striatum and SNpc of 3-month-old PITX3/GC mice. Scale bars: 10 µm. (C) Sagittal and (D) coronal sections showing TH (left, merged in red on the right) and GFP (central, merged in green on the right) immunoreactivity. Expression of GFP/GCaMP3 was only observed in mDA neurons and striatum of PITX3/GC double transgenic mice (arrowheads). Meanwhile, “leaky” expression of GFP was found in non-DA neurons in neocortex, cerebellum, and hippocampus of both tetO-GCaMP3 (−/GC) single and PITX3/GC double transgenic mice. Scale bars: 1 mm.

We generated PITX3-IRES2-tTA/tetO-GCaMP3 (PITX3/GC) inducible transgenic mice by crossbreeding PITX3-IRES2-tTA (PITX3/−) heterozygous knock-in mice expressing the tetracycline transactivator (tTA) under the control of mouse endogenous PITX3 promoter [12], with tetO-GCaMP3 (−/GC) transgenic mice that express GCaMP3 under the regulation of the tetracycline operator (tetO) (Fig. 1A). GCaMP3 expression in mDA neurons of PITX3/GC transgenic mice was visualized by co-immunostaining with specific antibodies against GFP and tyrosine hydroxylase (TH). The GFP signals appeared in the cytosol of soma and processes, in both substantia nigra pars compacta (SNpc) and ventral tegmental area (VTA) mDA neurons, as well as in dorsal striatal DA axons and terminals (Fig. 1B). Additional GFP immunoreactivity was detected in non-mDA neurons located in the posterior cerebral cortex, cerebellum, hippocampus, and other brain regions (Fig. 1C). The same expression pattern of GCaMP3 in non-mDA neurons was also observed in the brain of −/GC single transgenic mice, suggesting that tetO alone allows a tTA-independent “leaky” expression of GCaMP3 in non-mDA neurons, a phenomenon found previously using the same tetO expression vector [12]. Importantly, little or no GFP immunoreactivity was detected in striatum in the −/GC mouse slices (Fig. 1D). In contrast, strongly enhanced GCaMP3 expression was observed only in the somata and neurites of mDA neurons in PITX3/GC double transgenic mice (Fig. 1C, D). Thus, in the midbrain and striatum of this double transgenic line, the large majority of GCaMP3 expression is driven by the PITX-tTA driver in mDA neurons and axonal projections.

**Figure 2:**
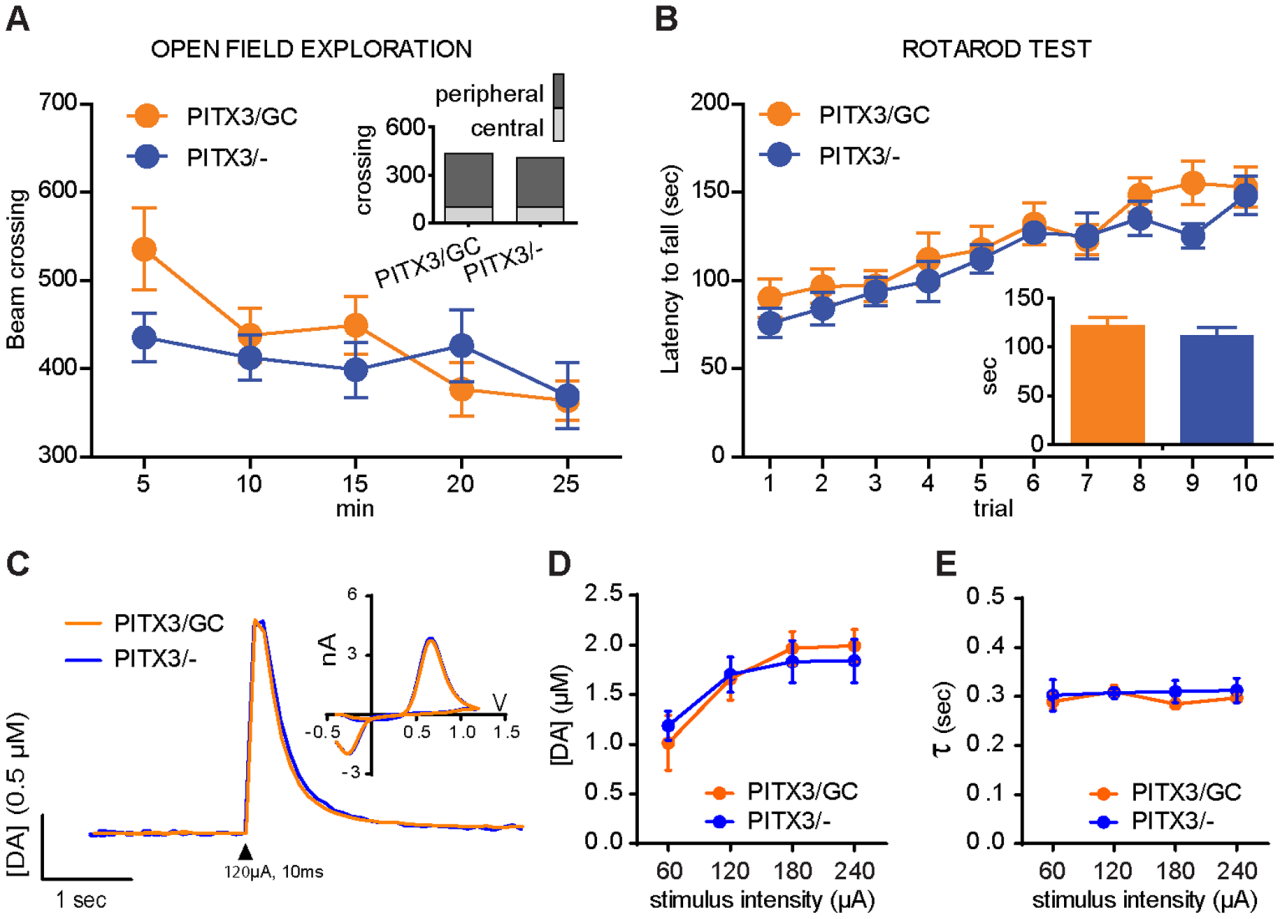
PITX3/GC transgenic mice display normal psychomotor phenotypes and DA transmission. (A) Open-field performance showed normal spontaneous exploration activity and regular thigmotaxis (shown in the inset) in PITX3-IRES2-tTA/tetO-GCaMP3 (PITX3/GC) double transgenic mice compared to PITX3-IRES2-tTA (PITX3/−) single transgenic mice. (B) Accelerating rotarod test performance of PITX3/GC mice was comparable to that of PITX3/− mice across a 10-trial session. (C) Representative DA traces (with cyclic voltammograms in inset) in response to single-pulse stimulation (black arrowhead: 120 µA, 10 ms, monophasic). The I/O curves from *ex vivo* fast-scan cyclic voltammetry experiments shows a normal peak evoked DA release (D) and tau (E) in PITX3/GC mice compared with PITX3/− single transgenic controls.

PITX3/GC transgenic mice developed normally and survived for the full expected life span (up to 18 months). The expression of GCaMP3 did not compromise spontaneous locomotion (ANOVA interaction: F_(4,64)_ = 01.88, p>0.05, genotype main factor: F_(1,64)_ = 0.69, p>0.05) or thigmotaxis (interaction: F_(1,16)_ = 1, p>0.05, genotype main factor: F_(1,16)_ = 0.79, p>0.05) in open-field tests (Fig. 2A), or motor skill performance on the accelerating rotarod test (interaction: F_(9,126)_ = 0.6, p>0.05; inset: genotype main factor: F_(1,126)_ = 1.21, p>0.05) (Fig. 2B). Moreover, fast scan cyclic voltammetry (FSCV) measurement in acute striatal slices revealed no differences in the amplitude or time course of stimulus-induced extracellular DA increases between PITX3/CG mice and their littermate PITX3/− controls (interaction: F_(3,24)_ = 0.29, p>0.05, genotype main factor: F_(1,24)_ = 0.02, p>0.05) (Fig. 2C, D). Comparable tau values from single exponential fits of the decay phase of the transients in slices from the two mouse lines were also observed, indicating that DA reuptake is unaltered by GCaMP3 expression (interaction: F_(3,24)_ = 0.18, p>0.05, genotype main factor: F_(1,24)_ = 0.87, p>0.05) (Fig. 2E). Thus, transgenic expression of GCaMP3 in mDA neurons does not alter crucial functions of nigrostriatal circuitry.

### Measurement of Presynaptic Ca^2+^Transients in mDA Neurons

**Figure 3:**
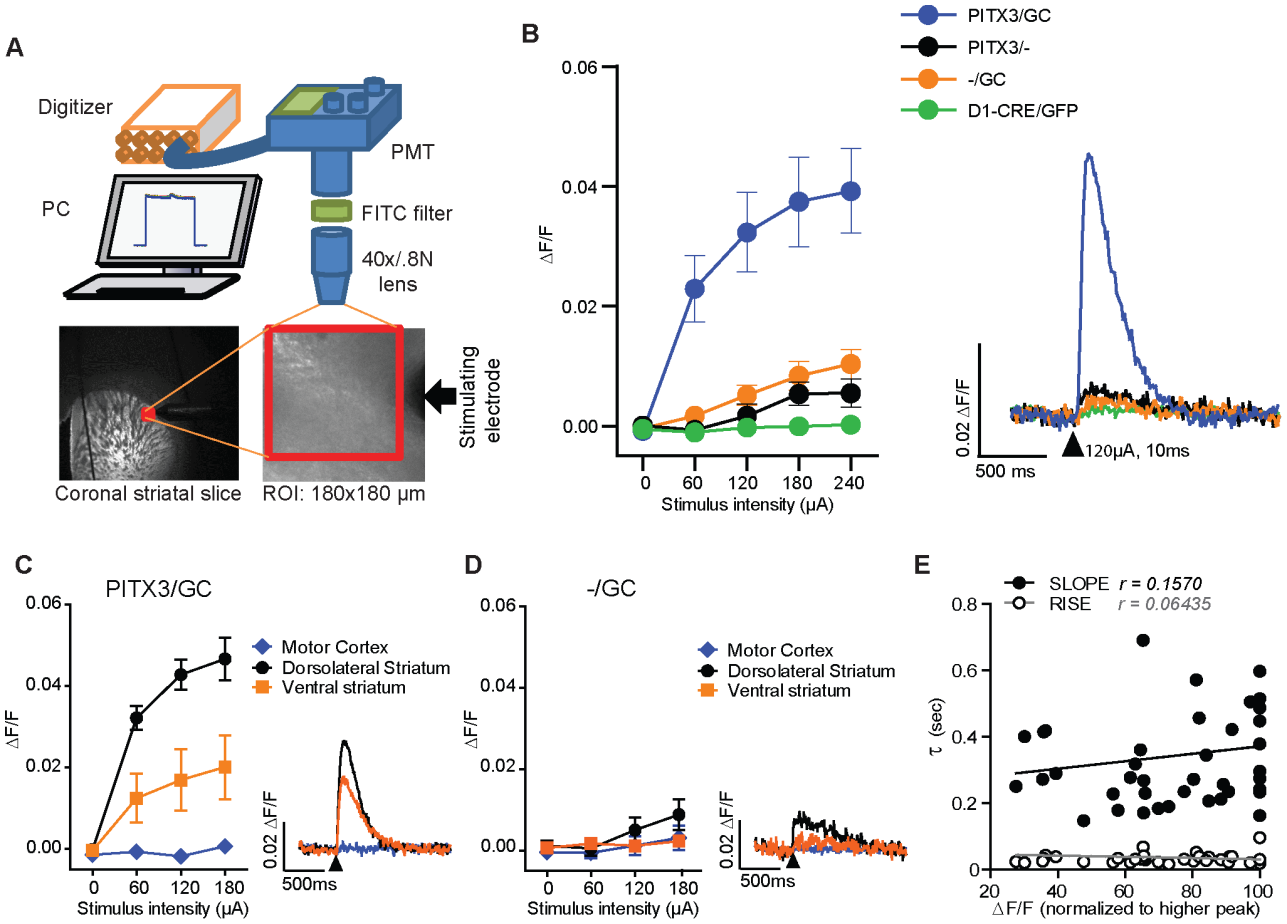
*Ex vivo* photometric measurement of Ca^2+^ transients in dopaminergic axons. (A) Cartoon illustrating the photometry setup (PMT: photomultiplier tube, FITC: Fluorescein isothiocyanate filter, PC: personal computer, ROI: region of interest). (B) I/O curves showing the peak amplitude of the fluorescence transient as a function of stimulus intensity for striatum in the four mouse lines. Representative traces are shown on the right. (black arrowhead: 120 µA, 10 ms, monophasic). (C) Comparison of I/O curves showing peak fluorescence transient amplitude as a function of stimulus intensity in different areas of coronal brain slices in PITX3-IRES2-tTA/tetO-GCaMP3 (PITX3/GC) mice. Dorsolateral striatum PreCaTs exhibited higher amplitudes compared with ventral striatum (Nucleus Accumbens, shell), with no detectable transients observed in the cortex (V layer of motor cortex). (D) tetO-GCaMP3 (−/GC) single transgenic mice showed minimal fluorescence changes after electrical stimulation in the striatum, about 10% compared to double transgenic mice at the highest stimulus intensities. (E) Scatterplots showing the time constant (τ) of the fluorescence transient rise and decay times in relation to peak amplitude of the PreCaT (normalized to maximum amplitude) for several individual dorsolateral striatum recordings. No significant correlations were observed.

Presynaptic Ca^2+^ dynamics from populations of mDA neuron afferents in dorsolateral striatum were measured with real-time acquisition in 250-µm thick coronal brain slices using photomultiplier tube (PMT)-based photometry (Fig. 3A). Single pulse intrastriatal electrical stimulation elicited readily detectable transient increases in fluorescence intensity in slices from PITX3/GC mice (expressed as ΔF/F) in the selected ROI (Fig. 3B, C). ROIs within motor cortex and ventral striatum (Nucleus Accumbens shell - NAs) were also selected from the same coronal slice (Bregma from +1.34 to +0.98 mm). ROIs in ventral striatum showed reduced transient amplitude compared to dorsolateral striatum, and transients were almost undetectable in cortex. This pattern corresponds well with the extent of DA innervation reported in these areas [21]. Slices from −/GC transgenic mice showed only small amplitude transients (∼10% of that observed in PITX3/GC mouse slices at stimulation intensities of 150–240 µA) (Fig. 3B, D). Meanwhile, PITX3/− mice showed very small amplitude transients (∼5% of those in PITX3/GC mouse slices) with a step-like shape (Fig. 3B), probably due to stimulation-induced changes in autofluorescence. At stimulation intensities of 120 µA or less, the transients in Pitx3/− and −/GC mice were barely detectable above background while transients in the PITX3/GC mice were near maximal (Fig. 3B). Thus, the 120- µA stimulation intensity was used in nearly all subsequent experiments to insure that calcium signals were nearly exclusively arising from populations of mDA afferents. No stimulus-induced transients were detected in striatal slices from mice expressing GFP driven by the dopamine D1 receptor promoter (Fig. 3B), confirming that transients are not derived from GFP or PMT sensor artifacts. In long lasting recording experiments, electrical stimulation (120 µA, 10 ms) was delivered every 30 sec, and stimulus-induced transients in dorsolateral striatum of the PITX3/GC mice were maintained with a ∼10–20% decrease in ΔF/F (compared to initial values) over 40 min of recording, demonstrating good photostability during the recording (also see Figure S2 in File S1).

The fluorescence transient onset and duration were analyzed by measuring the time constants (τ) of the initial rising phase of the transient, and the decay following the transient peak. The initial rise time of the transients was τ∼25 ms, while the range of decay τ values was 200–235 ms, without any significant correlation with amplitude of the transients (Fig. 3E. Rise time: Pearson r = 0.06435, p>0.05. Slope time: r = 0.1570, p>0.05).

### Presynaptic Fluorescent Transients Recorded in the Dorsolateral Striatum Are N- and P/Q-type Ca^2+^ Channel Dependent

**Figure 4:**
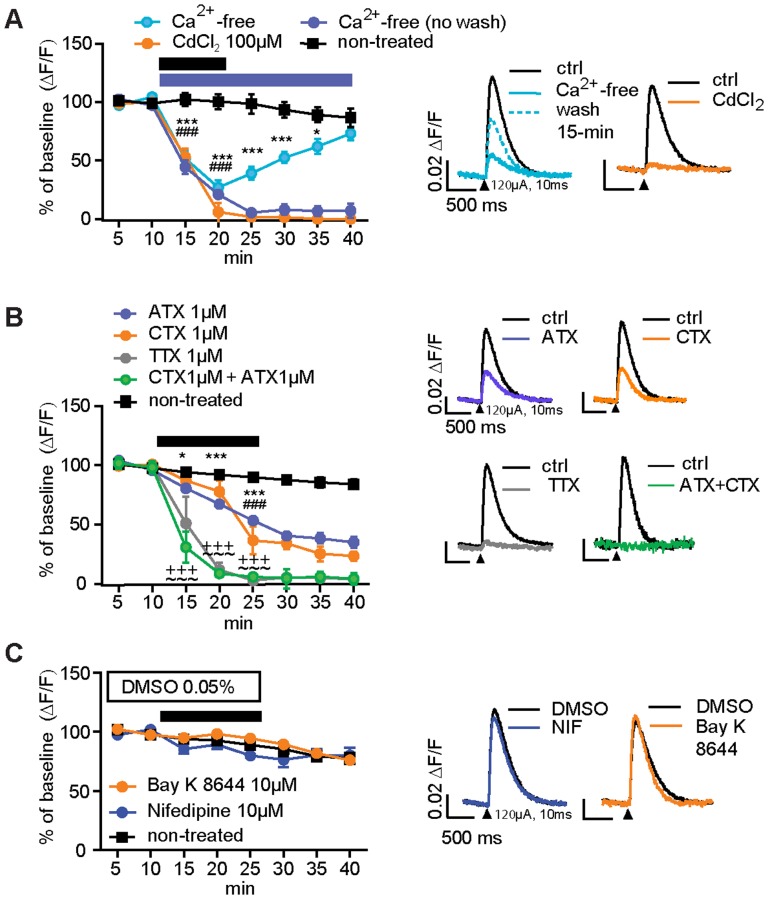
Ca^2+^ dependence and modulation of presynaptic fluorescence transients in mDA neurons. (A) Application of Ca^2+^-free aCSF significantly reduced PreCaTs in a reversible manner, while longer-lasting continuous application eliminated transients. Cadmium chloride (CdCl_2_, 100 µM), abolished PreCaTs (### = p<0.001). Representative transients are shown on the right. (B) N- and P/Q-type VGCC blockers partially reduced PreCaTs. P/Q-type (ω-agatoxin IVA, 1 µM) and N-type VGCC blockers (ω-conotoxin GVIA, 1 µM) reduced transients to ∼60% of the control value (* = p<0.05, *** = p<0.001) and ∼40% of control (### = p<0.001), respectively. Combined application of both toxins eliminated PreCaTs. (+++ = p<0.001). PreCaTs were also eliminated by TTX (1 µM; ∼∼∼ = p<0.001). Representative transients are shown at right. (C) L-type VGCC antagonist (Nifedipine, 1 µM) and agonist (Bay K 8644, 10 µM) had no significant effect on PreCaTs DA neurons. Representative traces are shown on the right (black arrowhead: 120 µA, 10 ms, monophasic).

Stimulus-induced fluorescence transients were eliminated in Ca^2+^-free aCSF after 15 min of perfusion (ANOVA interaction: F_(7,63)_ = 20.91, p<0.001)(Fig. 4A). Shorter duration exposure to Ca^2+^-free aCSF reduced transients, and subsequent application of normal [Ca^2+^]_o_ restored transients (interaction: F_(7,63)_ = 20.91, p<0.001)(Fig. 4A). Cadmium chloride (CdCl_2_ 100 µM), a nonselective voltage-gated Ca^2+^ channel (VGCC) blocker also eliminated transients (interaction: F_(7,63)_ = 62.22, p<0.001) (Fig. 4A). Thus, transients likely result from presynaptic Ca^2+^ entry. Striatal DA release is action potential- and Ca^2+^-dependent [22], and involves Ca^2+^ entry primarily through N- and P/Q-type Ca^2+^ channels [5], [23]. The I_Na_/action potential blocker TTX (1 µM) completely blocked stimulus-induced fluorescence transients in PITX3/GC mouse slices (interaction: F_(7,77)_ = 46.52, p<0.001)(Fig. 4B), but had no effect on transients observed in PITX/− mice (Figure S2 in File S1). Application of P/Q-type (ω-agatoxin IVA, ATX, 1 µM) and N-type (ω-conotoxin GVIA, CTX, 1 µM) Ca^2+^ channel blockers each reduced fluorescence transient amplitude to 40_–_60% below levels in untreated slices (interaction, ATX: F_(7,112)_ = 52.86, p<0.001. CTX: F_(7,84)_ = 27.17, p<0.001) (Fig. 4B). Combining these two blockers led to complete elimination of the fluorescent transient (interaction: F_(7,77)_ = 64.03, p<0.001)(Fig. 4C). In contrast, an agonist (Bay K 8644, 10 µM) and an antagonist (nifedipine, 10 µM) of L-type Ca^2+^ channels had no significant effects on transients (Fig. 4C). These results demonstrate that these stimulus-induced fluorescence transients were triggered by Ca^2+^ into the axons and axon terminals of mDA neurons. We will subsequently refer to these fluorescence transients as presynaptic Ca^2+^ transients (PreCaTs).

### Activation of Dopaminergic and Cholinergic Receptors Modulates PreCaTs of mDA Neurons

**Figure 5:**
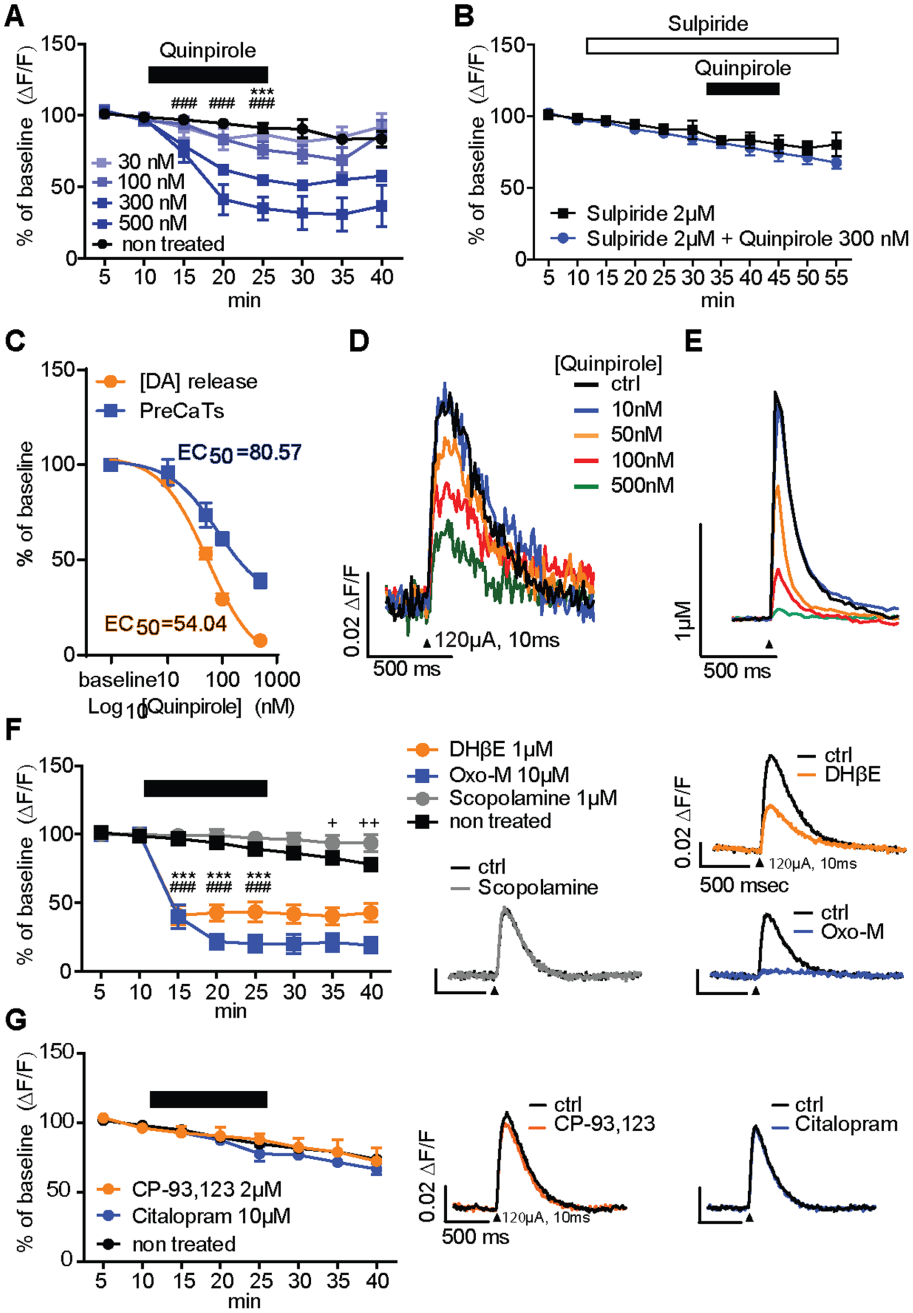
Pharmacological manipulations of PreCaTs in the dorsolateral striatum. (A) The D2AR agonist quinpirole significantly reduced PreCaT amplitude at 100 nM (*** = p<0.001), while 300 nM and 500 nM produced faster-developing and more complete inhibition (### = p<0.001). (B) Pre-exposure to the D2AR antagonist sulpiride (2 µM) prevented PreCaT reduction during perfusion of quinpirole. (C) Concentration-effect curves for quinpirole during simultaneous voltammetry (•) and photometry (▪) recordings. The agonist exhibits lower potency and efficacy for inhibiting PreCaTs compared to dopamine release. (D) Representative PreCaT traces induced by electrical stimulation (black arrowhead: 120 µA, 10 ms, monophasic), upon exposures to different concentration of quinpirole. (E) Representative DA traces simultaneously collected with FSCV and effects of the same quinpirole concentrations. (F) The α4β2 nAChR competitive antagonist DHβE (1 µM) produced a ∼50% inhibition of PreCaT peak amplitude (*** = p<0.001). The mAChR agonist oxotremorine-m (Oxo-M, 10 µM) reduced PreCaT peak amplitude to ∼25% of baseline levels (### = p<0.001). The mAChR antagonist scopolamine (1 µM) maintained baseline PreCaTs above levels normally observed in non-treated controls, especially late in recordings (+ = p<0.05,++ = p<0.01). (G) The selective serotonin reuptake inhibitor citalopram (10 µM) and the of 5-HT1B receptor agonist CP-93,129 (2 µM) failed to alter the PreCaTs in mDA neurons. Representative traces are shown on the right (black arrowhead: 120 µA, 10 ms, monophasic).

The dopamine D2 autoreceptor (D2AR) agonist quinpirole produced a concentration-dependent inhibition of PreCaTs in striatal slices from PITX3/GC mice (ANOVA interactions: 100 nM: F_(7,49)_ = 4.71, p<0.001. 300 nM: F_(7,42)_ = 22.2, p<0.001. 500 nM: F_(7,42)_ = 13.43, p<0.001) (Fig. 5A). This effect was blocked by pre-exposure to the D2-type receptor antagonist sulpiride (Fig. 5B), indicating that this inhibition likely involves D2ARs. We performed simultaneous photometric and FSCV recordings to examine the D2 agonist quinpirole effects on PreCaTs and DA release evoked by the same afferent stimuli. Notably, the potency of quinpirole appeared to be lower by for inhibition of PreCaTs in comparison to its inhibitory effect on DA release. (Fig. 5C, D, E). Exposure of PITX3/GC striatal slices to the α4β2 competitive nicotinic acetylcholine receptor (nAChR) antagonist dihydro-β-erythroidin (DHβE, 1 µM) produced a ∼50% decrease in PreCaT amplitude compared to non-treated controls (interaction: F_(7,56)_ = 39.56, p<0.001) (Fig. 5F). The muscarinic acetylcholine receptor (mAChR) agonist oxotremorine-m (Oxo-M, 10 µM) produced a decrease of ∼75% in PreCaT amplitude (interaction: F_(7,42)_ = 43, p<0.001) (Fig. 5F). In the presence of the mAChR competitive antagonist scopolamine (1 µM), PreCaT amplitude was maintained near baseline levels, which was significantly different from the rundown observed under control conditions (interaction: F_(7,98)_ = 4.26 p<0.001) (Fig. 5F). Our work suggests a direct influence of DARs and AChRs on presynaptic Ca^2+^ in dorsostriatal DA fibers.

### Serotonin Signaling Does Not Affect the PreCaTs in the mDA Axons and Terminals

Increasing endogenous serotonin (5-HT) levels using the selective serotonin reuptake inhibitor (SSRI) citalopram (10 µM), or directly activating 5-HT1B receptors using the agonist CP-93,129 (2 µM) did not affect PreCaTs in mDA inputs to striatum (Fig. 5G). Thus, our experiments did not reveal evidence of any direct serotonergic modulation of presynaptic Ca^2+^ that would contribute to inhibition of DA release by this monoamine neuromodulator.

### Simultaneous Paired Recordings of PreCaTs with [DA] Release and Extracellular Field Potentials

**Figure 6:**
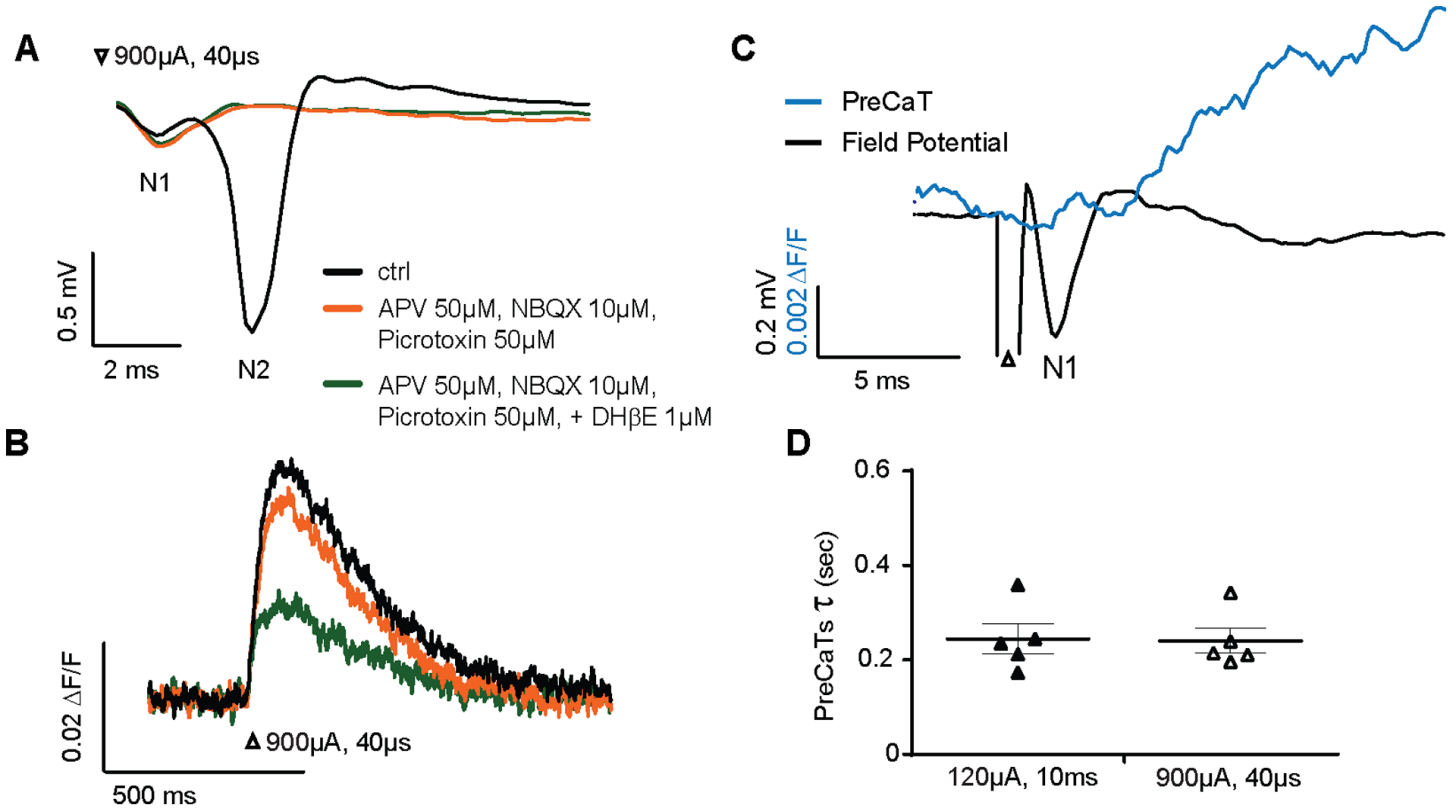
Simultaneous PreCaT and extracellular field potential recordings. (A) Representative field potential recordings and simultaneously measured PreCaT traces (B) induced by electrical stimulation (white arrowhead: 900 µA, 40 µs, monophasic). Effects of application of a cocktail of antagonists of ionotropic glutamate and GABA receptors ((2R)-amino-5-phosphonovaleric acid (APV, 50 µM); 2,3-dihydroxy-6-nitro-7-sulfamoyl-benzo[f]quinoxaline-2,3-dione (NBXQ, 10 µM)) are also shown. Perfusion of the drug cocktail for 10 min eliminated the synaptically driven postsynaptic component (N2) of the field potential, leaving intact the directly electrical stimulation-driven fiber volley component (N1). A marginal decrease in the PreCaT was observed following application of the antagonist cocktail. Subsequent application of the nAChR competitive antagonist DHβE (1 µM) produced strong inhibition of the PreCaT. (C) Traces from simultaneous record of the N1/fiber volley and PreCaT. The time course of presynaptic Ca2+ increases in relation to single afferent action potentials generated in a population of fibers. (D) PreCaTs time constant of slopes calculated from two different stimulation conditions (FSCV vs field potential double recording sessions). No significant difference was observed.

To determine if PreCaTs were evoked by stimuli that produce single presynaptic action potentials, we also performed simultaneous photometry and extracellular field potential recordings. In these experiments, we used shorter stimulation pulses (40 µs) that reliably evoked single fiber volleys (N1 field potential component, non-synaptically driven potentials in afferent fibers generated by stimulation), and synaptically driven population spikes (N2 field potential component), as previously observed [24] (Fig. 6A). The same stimulation simultaneously evoked PreCaTs with duration similar to that observed with the longer-duration stimuli (Fig. 6B, C, D). Application of a cocktail of ionotropic glutamate receptor antagonists (NBQX, 10 µM, APV, 50 µM) and a GABA_A_ receptor antagonist (picrotoxin, 50 µM) eliminated the N2, but not N1 field potential component within ∼10 min, as expected (Fig. 6A), but produced only a marginal decrease in PreCaT amplitude (Figure 6B). We took advantage of our ability to simultaneously record the N1/fiber volley and PreCaT to compare the time course of presynaptic Ca^2+^ increases in relation to single afferent action potentials generated in a population of fibers. Figure 6C shows that the PreCaT onset occurs with a very short latency after the peak of the fiber volley/N1 component of the field potential, as one would expect for a response that reflects Ca2+ entry directly driven by presynaptic afferent activation. The increase in presynaptic Ca2+ occurs in close temporal relationship to N1, and before the expected onset of synaptic responses, as observed in past photometric recordings of PreCaTs [25].

## Discussion

Recent improvements in GECI kinetic properties and sensitivity have resulted in reporters that allow for ever-improving monitoring of activity-driven Ca^2+^ changes [26]. Investigators can now begin to use these tools to study the activation of DA terminals but also to probe pathological changes in models of neurological disorders. While GCaMP3 lacks the sensitivity of more recently developed GECIs [26], our findings indicate that it is clearly usable for detection of Ca^2+^ transients from populations of densely packed presynaptic elements even under stimulation conditions where only one action potential is elicited. This approach allowed us to investigate PreCaTs using real-time measurements in DA axons/terminals within striatal slices, which would be very difficult using Ca^2+^-sensitive dye-based techniques. The characteristics of these Ca^2+^ transients corresponded with known mechanisms of DA release. While the photometric method does not allow for measurement of Ca^2+^ specifically within the active zones of DA terminals, the strong relationship between factors that underlie and modulate the PreCaTs and DA release suggests that we can learn a great deal about presynaptic functions related to DA dynamics using this approach. Our reported effects of ionic manipulation and toxins strongly support the conclusion that the stimulus-induced increases in fluorescence measured in striatal slices arise from presynaptic action potential-dependent Ca^2+^ influx via mechanisms implicated in DA release.

While we report kinetic parameters of the PreCaTs, we are aware that such parameters do not always faithfully report the kinetics of intracellular free Ca^2+^ concentration changes that occur naturally in the absence of a Ca^2+^ indicator. The onset and duration of Ca^2+^ transients are affected not only by the effective Ca^2+^ flow in and out of terminals, but they also reflect GCaMP binding and kinetic properties. GCaMP3 in particular showed slower kinetics compared to the most recent generation GCaMP constructs [26], but still within the range of decay times of PreCaTs observed with the most common Ca^2+^ indicators [27]. In the absence of robust data on the true kinetics of PreCaTs in dopaminergic neurons, the time constants measured in our PITX3/GC mice might be considered as a limited estimation of rise and clearance of Ca^2+^ within the terminals. It is reassuring that DA release was not altered by the presynaptic expression of GCaMP3, as this finding indicates that the GECI is not buffering presynaptic Ca^2+^ at levels/location that would disrupt sensitive excitation/secretion coupling mechanisms. The observation that Ca^2+^ kinetics were not greatly altered at different stimulus durations or intensities indicates that GCaMP3 is reporting a relatively uniform process of increased presynaptic Ca^2+^ even when the number of afferents stimulated is greatly increased.

An important feature of DA transmission in striatum is presynaptic autoinhibition, in which dopamine modulates its own release and synthesis through presynaptic D2AR located at DA axon terminals in the striatum [28], [29]. Activation of D2AR or other G_i/o_ subtype G-protein-coupled receptors (GPCRs) may reduce neurotransmitter release through inhibition of presynaptic Ca^2+^ entry, a common mechanism underlying presynaptic GPCR effects [30], [31]. Data from simultaneous dual FSCV/photometry recordings presented in this study indicate different potencies of D2AR agonists for inhibition of PreCaTs and DA release. One explanation for this finding is that inhibition of release could involve multiple signaling pathways, some of which may require lower receptor occupancy or more post-receptor amplification than inhibition of Ca^2+^ channels. Alternatively, given the strong cooperativity in the relationship between presynaptic Ca^2+^ levels and vesicle fusion [32], small decreases in Ca^2+^ transients may have large effects on DA release.

Interactions between striatal ACh and dopamine influence many aspects of striatal function and related behaviors [33]. ACh stimulates DA release through nicotinic acetylcholine receptors (nAChRs) on DA axons [34], mainly via β2-subunit-containing receptors [35], [36]. Activation of cholinergic striatal interneurons directly stimulates DA release [37]. Meanwhile, muscarinic acetylcholine receptors (mAChRs) modulate DA release in a number of ways [38], [39], [40]. Our work shows a direct influence of AChRs on presynaptic Ca^2+^ in mDA neurons. Single-stimulus-evoked presynaptic Ca^2+^ rises in mDA neurons are suppressed when β2-nAChR on DA axons are directly antagonized. This finding is consistent with the known role of these receptors in DA release in striatal slices [37], [38], [41]. Activation of mACh autoreceptors decreased PreCaTs in the dopaminergic afferents, and blockade of mACh appeared to relieve endogenous cholinergic tone that inhibits PreCaTs. These actions are good candidate mechanisms for the known AChR effects on dopamine release.

In brain areas containing dopaminergic terminals, various serotonin (5-HT) receptors have been shown to modulate dopamine release [42]. In striatum, dopamine release is reduced by presynaptic 5-HT1B heteroreceptors [43]. We did not observe any effect of 5-HT on PreCaTs in dopaminergic neurons. It is possible that these receptors have indirect effects that decrease DA release, perhaps involving disinhibition of GABAergic interneurons, as described in the dorsal striatum of 5-HT1B knockout mice using in vivo microdialysis [44].

Finally, we were also able to make simultaneous field potential and PreCaT recordings with an appreciable noise to signal ratio. From these experiments we can clearly see that PreCaTs are driven by, and temporally associated with, single action potentials driven in populations of afferent fibers.

In conclusion, combining GCaMP expression with photometry in brain slices allowed us to examine the molecular basis and modulation of PreCaTs in mDA neurons, and should make it possible to measure these presynaptic responses in a variety of afferent inputs made by projection neurons. Aberrant striatal dopaminergic transmission contributes to various neurological disorders. Although pathological changes and motor dysfunction that characterize diseases like Parkinson’s disease are well documented, the mechanism(s) responsible for dysfunction of DA neurons and terminals have yet to be clearly established. We can use the present approach to study the involvement of Ca^2+^ signaling simultaneously with other conventional striatal physiology techniques and during the progression of severe neuropathology of the DA system.

## Supporting Information

File S1 Contains the following files: **Figure S1.** Description of raw data and mathematical correction for sampling epochs. Related to Data Analysis Procedures (A) Raw data traces showing the total fluorescence (*F*) and stimulus-induced change in fluorescence (*ΔF*) during several trials in a single slice from a PITX3-IRES2-tTA/tetO-GCaMP3 (PITX3/GC) mouse. Box indicates segment of the traces used for linear regression and baseline normalization in the entire trace (left) and with expanded axes (right). Each sweep shows a 6-sec window in which the shutter was open (excitation light exposure) for 5 sec. Two sec after the opening of the shutter, electrical stimulation was delivered, with a consequent increase of the fluorescence recorded by the PMT. (B) The boxed portion of averaged sweeps (2 sec total) was exported and analyzed. For baseline normalization, the peak of the time period containing the stimulus-induced transient was deleted and linear regression was performed on the remaining data points. A linear function was calculated and values at each point on this line were estimated. Each point in the original raw exported data was divided by the fit-generated estimated value at the same time point to obtain the normalized baseline value across the entire 2 sec period. (C) The final result of this baseline normalization is shown for the sweeps in (A) and (B). (D) UV light interfered with basal current recorded by FSCV electrode, producing an increase in current proportional to the intensity of the light used. (E) Representative DA traces (with cyclic voltammograms in inset) and color plots showing responses to single-pulse stimulation (black arrowhead: 120 µA, 10 ms, monophasic) with exposure to light from the mercury burner (20% intensity). Note that the light-induced current is strongest at electrode potentials more positive than the voltages associated with DA oxidation. In the same fashion described above for correction of photometric correction traces shown in panel B, we corrected for the light-induced change voltammetric current in the boxed portion of the DA transient. (F) shows the signals before and after linear regression calculation and subtraction, and compared with a light OFF trace sample. Note the lack of effect of light exposure or the correction procedure on the DA transient. **Figure S2.** Characterization of stimulus-induced fluorescence transients in PITX3/GC, PITX3/− and −/GC mouse striatal slices. (A) Graphs showing effects of 4-Aminopyridine (4-AP) and (B) Tetrodotoxin (TTX), as well as with representative traces (C) of PreCaTs in single and double transgenic mice. Transients in double PITX3-IRES2-tTA/tetO-GCaMP3 (PITX3/GC) and single tetO-GCaMP3 (−/GC) transgenic mice were increased by the potassium channel inhibitor 4-AP and decreased by inhibition of the firing of action potentials with TTX. Note that PITX3-IRES2-tTA (PITX3/−) mouse slices expressed a small, drug-insensitive transient similar to the transients observed in slices from the other two lines after TTX application. This step-like response is most likely mainly a consequence of stimulation-induced changes in autofluorescence easily detected against a low fluorescence background (D). In fact, no such transient was detectable in slices from D1-cre/GFP transgenic mice, presumably due to their relatively high background fluorescence (as reported in Fig. 3B). (E) Comparison of different inter-stimulus interval (ISI) effects on stability of PreCaTs. Stimulation with 5-min and 3-min ISIs revealed a minor (7–15%) decrease in current amplitude that overlapped with that observed during to our standard 30-sec ISI condition (10–20% decrease). Thus, the time-dependent loss of signal is probably due to a combination of both GFP-photobleaching and loss of signal from some afferents. As such, we routinely used the 30-sec ISI to obtain more time points for averaging traces that reduced interference from noise in the PreCaT calculations. Note that we included no-treatment conditions for experiments with drug exposure and prolonged recordings (e.g. Fig. 4 and 5) to control for the time-dependent loss of signal.
(PDF)
