# Supplementary material for: Optogenetic Measurement of Presynaptic Calcium Transients Using Conditional Genetically Encoded Calcium Indicator Expression in Dopaminergic Neurons
Source: PLoS One. 2014 Oct 31;9(10):e111749. doi: 10.1371/journal.pone.0111749 (PMC4216119; doi:10.1371/journal.pone.0111749)
Supplement: File S1 — Contains the following files: Figure S1. Description of raw data and mathematical correction for sampling epochs. Related to Data Analysis Procedures (A) Raw data traces showing the total fluorescence (F) and stimulus-induced change in fluorescence (ΔF) during several trials in a single slice from a PITX3-IRES2-tTA/tetO-GCaMP3 (PITX3/GC) mouse. Box indicates segment of the traces used for linear regression and baseline normalization in the entire trace (left) and with expanded axes (right). Each sweep shows a 6-sec window in which the shutter was open (excitation light exposure) for 5 sec. Two sec after the opening of the shutter, electrical stimulation was delivered, with a consequent increase of the fluorescence recorded by the PMT. (B) The boxed portion of averaged sweeps (2 sec total) was exported and analyzed. For baseline normalization, the peak of the time period containing the stimulus-induced transient was deleted and linear regression was performed on the remaining data points. A linear function was calculated and values at each point on this line were estimated. Each point in the original raw exported data was divided by the fit-generated estimated value at the same time point to obtain the normalized baseline value across the entire 2 sec period. (C) The final result of this baseline normalization is shown for the sweeps in (A) and (B). (D) UV light interfered with basal current recorded by FSCV electrode, producing an increase in current proportional to the intensity of the light used. (E) Representative DA traces (with cyclic voltammograms in inset) and color plots showing responses to single-pulse stimulation (black arrowhead: 120 µA, 10 ms, monophasic) with exposure to light from the mercury burner (20% intensity). Note that the light-induced current is strongest at electrode potentials more positive than the voltages associated with DA oxidation. In the same fashion described above for correction of photometric correction traces shown in panel B, we [file pone.0111749.s001.pdf]

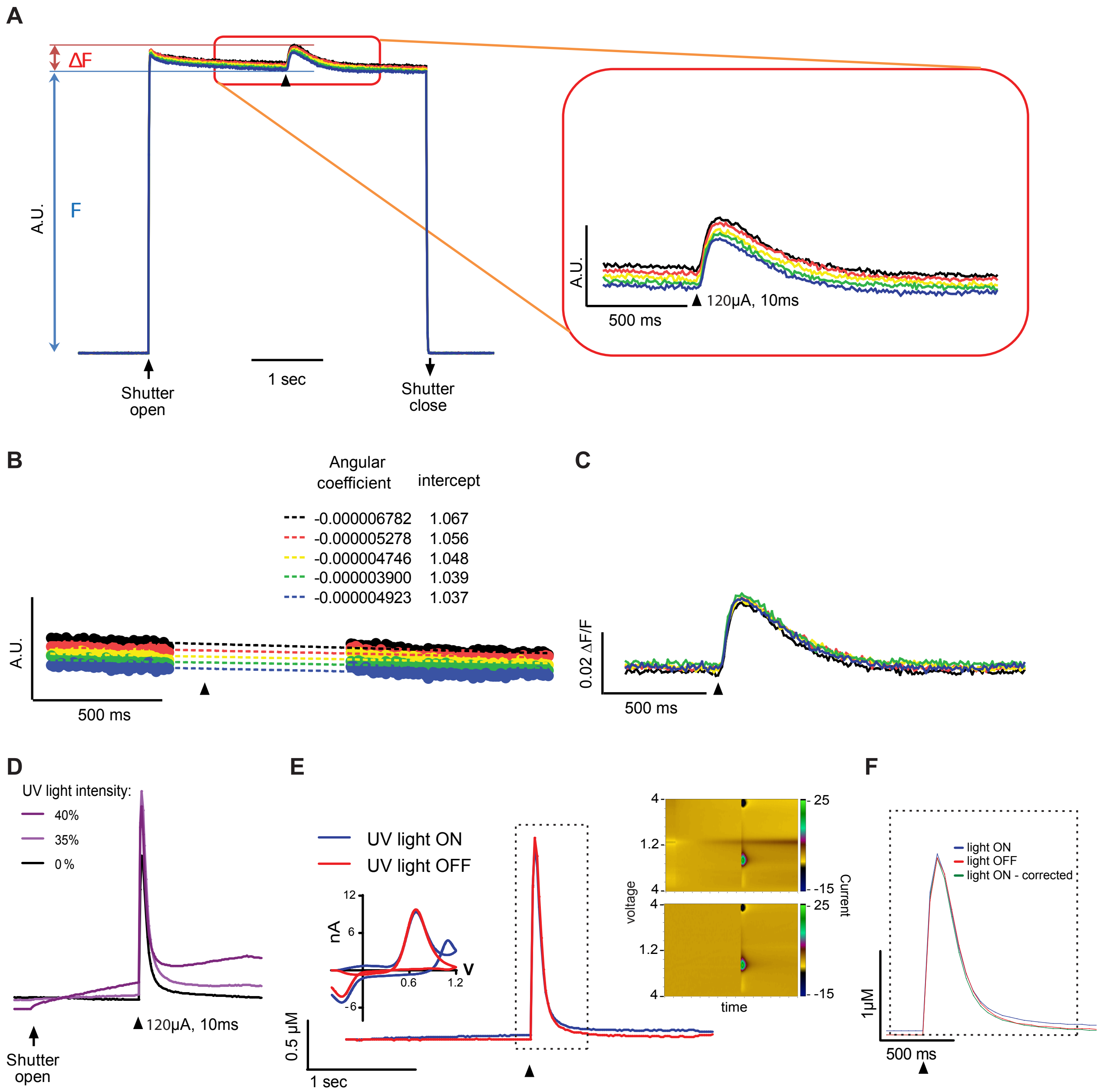


**Figure S1. Description of raw data and mathematical correction for sampling epochs.** Related to Data Analysis Procedures (A) Raw data traces showing the total fluorescence (*F*) and stimulus-induced change in fluorescence (*ΔF*) during several trials in a single slice from a PITX3-IRES2-tTA/tetO-GCaMP3 (PITX3/GC) mouse. Box indicates segment of the traces used for linear regression and baseline normalization in the entire trace (left) and with expanded axes (right). Each sweep shows a 6-sec window in which the shutter was open (excitation light exposure) for 5 sec. Two sec after the opening of the shutter, electrical stimulation was delivered, with a consequent increase of the fluorescence recorded by the PMT. (B) The boxed portion of averaged sweeps (2 sec total) was exported and analyzed. For baseline normalization, the peak of the time period containing the stimulus-induced transient was deleted and linear regression was performed on the remaining data points. A linear function was calculated and values at each point on this line were estimated. Each point in the original raw exported data was divided by the fit-generated estimated value at the same time point to obtain the normalized baseline value across the entire 2 sec period. (C) The final result of this baseline normalization is shown for the sweeps in (A) and (B). (D) UV light interfered with basal current recorded by FSCV electrode, producing an increase in current proportional to the intensity of the light used. (E) Representative DA traces (with cyclic voltammograms in inset) and color plots showing responses to single-pulse stimulation (black arrowhead, 120 µA, 10 ms, monophasic) with exposure to light from the mercury burner (20% intensity). Note that the light-induced current is strongest at electrode potentials more positive than the voltages associated with DA oxidation. In the same fashion described above for correction of photometric correction traces shown in panel B, we corrected for the light-induced change voltammetric current in the boxed portion of the DA transient. (F) shows the signals before and after linear regression calculation and subtraction, and compared with a light OFF trace sample. Note the lack of effect of light exposure or the correction procedure on the DA transient.

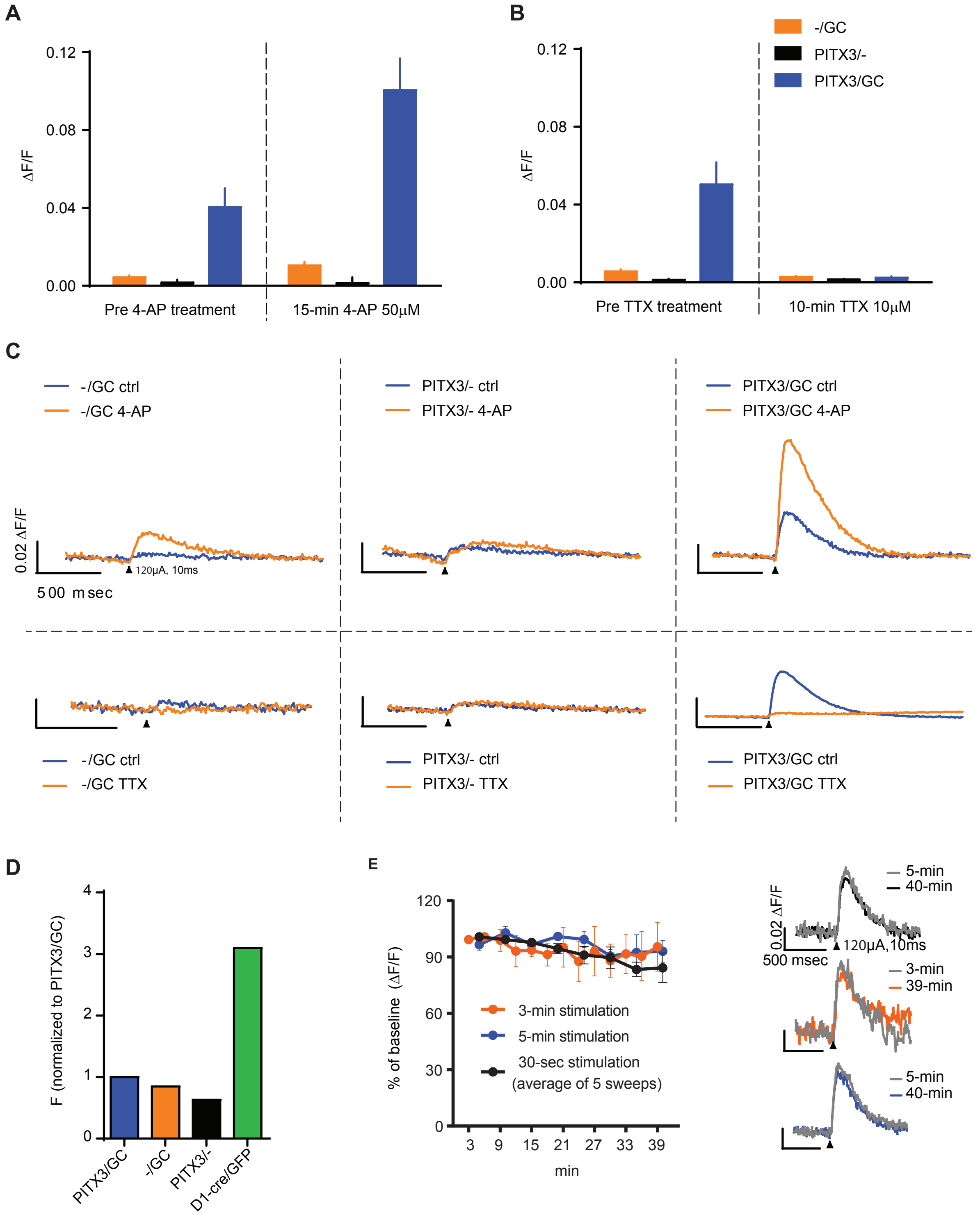


Figure S2. Characterization of stimulus-induced fluorescence transients in PITX3/GC, PITX3/- and -/GC mouse striatal slices. (A) Graphs showing effects of 4-Aminopyridine (4-AP) and (B) Tetrodotoxin (TTX), as well as with representative traces (C) of PreCaTs in single and double transgenic mice. Transients in double PITX3-IRES2-tTA/tetO-GCaMP3 (PITX3/GC) and single tetO-GCaMP3 (-/GC) transgenic mice were increased by the potassium channel inhibitor 4-AP and decreased by inhibition of the firing of action potentials with TTX. Note that PITX3-IRES2-tTA (PITX3/-) mouse slices expressed a small, drug-insensitive transient similar to the transients observed in slices from the other two lines after TTX application. This step-like response is most likely mainly a consequence of stimulation-induced changes in autofluorescence easily detected against a low fluorescence background (D). In fact, no such transient was detectable in slices from D1-cre/GFP transgenic mice, presumably due to their relatively high background fluorescence (as reported in Fig. 3B). (E) Comparison of different inter-stimulus interval (ISI) effects on stability of PreCaTs. Stimulation with 5-min and 3-min ISIs revealed a minor (7-15%) decrease in current amplitude that overlapped with that observed during to our standard 30-sec ISI condition (10-20% decrease). Thus, the time-dependent loss of signal is probably due to a combination of both GFP-photobleaching and loss of signal from some afferents. As such, we routinely used the 30-sec ISI to obtain more time points for averaging traces that reduced interference from noise in the PreCaT calculations. Note that we included no-treatment conditions for experiments with drug exposure and prolonged recordings (e.g. Fig. 4 and 5) to control for the time-dependent loss of signal.
